# Jasmonic Acid-Induced VQ-Motif-Containing Protein OsVQ13 Influences the OsWRKY45 Signaling Pathway and Grain Size by Associating with OsMPK6 in Rice

**DOI:** 10.3390/ijms20122917

**Published:** 2019-06-14

**Authors:** Yuya Uji, Keita Kashihara, Haruna Kiyama, Susumu Mochizuki, Kazuya Akimitsu, Kenji Gomi

**Affiliations:** Plant Genome and Resource Research Center, Faculty of Agriculture, Kagawa University, Miki, Kagawa 761-0795, Japan; ijuayuy@gmail.com (Y.U.); 8743002z@stu.kagawa-u.ac.jp (K.K.); s18g621@stu.kagawa-u.ac.jp (H.K.); motti245@ag.kagawa-u.ac.jp (S.M.); kazuya@ag.kagawa-u.ac.jp (K.A.)

**Keywords:** MAP kinase, jasmonate, rice bacterial blight, salicylic acid, grain development

## Abstract

Jasmonic acid (JA) is a plant hormone that plays an important role in the defense response and stable growth of rice. In this study, we investigated the role of the JA-responsive valine-glutamine (VQ)-motif-containing protein OsVQ13 in JA signaling in rice. OsVQ13 was primarily located in the nucleus and cytoplasm. The transgenic rice plants overexpressing *OsVQ13* exhibited a JA-hypersensitive phenotype and increased JA-induced resistance to *Xanthomonas oryzae* pv. *oryzae* (*Xoo*), which is the bacteria that causes rice bacterial blight, one of the most serious diseases in rice. Furthermore, we identified a mitogen-activated protein kinase, OsMPK6, as an OsVQ13-associating protein. The expression of genes regulated by OsWRKY45, an important WRKY-type transcription factor for *Xoo* resistance that is known to be regulated by OsMPK6, was upregulated in *OsVQ13*-overexpressing rice plants. The grain size of *OsVQ13*-overexpressing rice plants was also larger than that of the wild type. These results indicated that OsVQ13 positively regulated JA signaling by activating the OsMPK6–OsWRKY45 signaling pathway in rice.

## 1. Introduction

Rice (*Oryza sativa* L.) is a major crop in the world, and decreased crop yields caused by pathogen attacks is a serious problem in rice farming. Many studies have shown that rice plants have developed complex defense systems to protect themselves against various pathogens. Among the defense systems, two plant hormones, jasmonic acid (JA) and salicylic acid (SA), are important signaling compounds that help to regulate the expression of defense-related genes in rice [1]. Treatment with benzothiadiazole (BTH), an SA analog, upregulates many pathogenesis-related (PR) genes and defense-related transcription factors (TFs) in rice and enhances resistance to rice bacterial blight and rice blast caused by *Xanthomonas oryzae* pv. *oryzae* (*Xoo*) and *Pyricularia oryzae*, respectively, which are both hemibiotrophic pathogens causing two of the most serious rice diseases, rice bacterial blight and rice blast, respectively [2]. The WRKY-type TFs, identified as a TF family containing a conserved WRKY domain [3], have important roles in rice defense responses. As one of the WRKY-type TFs, OsWRKY45 plays a crucial role in the BTH-mediated defense response against *Xoo* and *P. oryzae* [2,4]. OsNPR1, a rice homologue to *Arabidopsis* NON-EXPRESSOR OF PATHOGENESIS-RELATED GENES1 (AtNPR1) [5], acts as a positive regulator of SA signaling and is involved in SA-mediated defense response in rice [6,7,8].

JA also plays an important role in the defense response against *Xoo*. Prior treatment with *Xoo*-derived cellulase (ClsA) and lipase/esterase (LipA) increases a rice plant’s resistance to subsequent *Xoo* infection and upregulates the expression of JA-biosynthetic and JA-responsive genes [9]. The JA-upregulated rice jasmonate ZIM domain (JAZ) protein, OsJAZ8, interacts with the F-box protein CORONATINE INSENSITIVE 1 (COI1), which is the primary JA receptor, and acts as a repressor of the JA response, thus negatively regulating the expression of JA-responsive defense-related genes and resistance to *Xoo* [10]. OsWRKY45-2 is involved in the JA-mediated resistance to *Xoo* [11]. Activation of the Cysteine3Histidine (CCCH)-type zinc-finger DNA-binding protein has been reported to induce JA-mediated resistance to *Xoo* in rice [12]. The basic helix–loop–helix (bHLH)-type TF OsMYC2, which is the rice homologue of AtMYC2, positively regulates the JA-mediated defense response against *Xoo* in rice [13]. OsNINJA1, which is the rice homologue of *Arabidopsis* NOVEL INTERACTOR OF JAZ (AtNINJA) [14], acts as a negative regulator of the OsMYC2-mediated defense response against *Xoo* in rice [15]. JA-induced volatiles such as monoterpenes and sesquiterpenes act as antibacterial or signaling compounds in the defense response against *Xoo* [16,17,18,19,20]. Of these JA-induced monoterpenes, linalool functions as a signal molecule to induce the upregulation of defense-related genes in rice [17]. In addition, (*E*)-nerolidol and γ-terpinene exhibit antimicrobial activities against *Xoo* [19,20]: γ-terpinene induces antibacterial activity against *Xoo* by damaging the bacterial plasma membrane [19]. The JA-induced accumulation of some volatiles is regulated by OsJAZ8 [17,18]. These results suggest that the JA signaling pathway is necessary for inducing rice defense systems against *Xoo*.

Recent studies have reported that JA-responsive plant-specific valine-glutamine (VQ) (FxxxVQxLTG)-motif-containing proteins have been identified in many plant species [21,22,23,24]. In *Arabidopsis*, JASMONATE-ASSOCIATED VQ MOTIF GENE1 (JAV1, known as AtVQ22) acts as a negative regulator of JA-mediated plant defense, operating against necrotrophic pathogens and herbivorous insects [25]. MITOGEN-ACTIVATED PROTEIN KINASE4 SUBSTRATE1 (MKS1, also known as AtVQ21) is required for the activation of SA-dependent defense [26,27]. In addition, some VQ-motif-containing proteins have been shown to interact with numerous WRKY TFs [21]. AtVQ23 interacts with AtWRKY33, a key regulator of plant defense against necrotrophic pathogens [26,28,29]. These results suggest that VQ-motif-containing proteins act as modulators in JA- and SA-mediated plant defense. The rice genome contains 39 VQ-motif-containing protein family genes, some of which respond to pathogen attacks [22,30]. However, studies on VQ-motif-containing proteins in JA-mediated defense signaling in rice are lacking. We recently identified a few JA-responsive VQ-motif-containing genes in rice through a microarray analysis [10]. In the current study, we investigated the role of the JA-responsive rice VQ-motif-containing protein OsVQ13 in the JA-mediated defense response in rice. We also provide evidence regarding an OsVQ13-associating protein, which acts as a key regulator of OsWRKY45.

## 2. Results

### 2.1. Properties of OsVQ13

**Figure 1:**
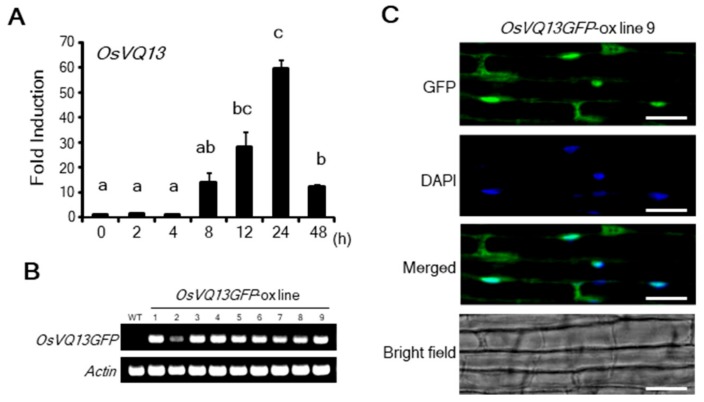
Jasmonic acid (JA)-induced expression and subcellular localization of OsVQ13. (**A**) Expression levels of *OsVQ13* in response to JA. Total RNA was extracted at the indicated time points after 100 µM of JA treatment. Values are means ± SE. Data were analyzed using Tukey’s HSD test (*n* = 4 for each genotype). Bars with different letters are significantly different at *p* < 0.05. (**B**) Reverse transcription (RT)-PCR analysis of *OsVQ13GFP* and *actin* expression in wild-type (WT) and *OsVQ13GFP*-overexpressing rice plants (*OsVQ13GFP*-ox; lines 1–9). (**C**) Subcellular localization of OsVQ13. The green fluorescent protein (GFP) signal in the root tissue of *OsVQ13GFP*-ox (line 9) was visualized by fluorescence microscopy. Nuclear localization of OsVQ13GFP was confirmed by 4′,6-diamidino-2-phenylindole (DAPI) staining. Bars = 10 µm.

We carried out reverse transcription-quantitative PCR (RT-qPCR) analysis of *OsVQ13* to investigate its expression in response to JA treatment. The expression of *OsVQ13* reached its maximum level after 24 h of JA treatment (Figure 1A). To determine the subcellular localization of OsVQ13, we generated transgenic rice plants overexpressing the OsVQ13 green fluorescent protein (GFP) fusion protein (*OsVQ13GFP*-ox) and confirmed the expression of the transgene through RT-PCR (Figure 1B). The GFP signal in the root tissue of *OsVQ13GFP*-ox line 9 was observed by fluorescence microscopy. As shown in Figure 1C, the GFP fluorescent signals were detected in the nucleus and the cytoplasm, indicating that OsVQ13 was localized in these specific locations.

### 2.2. Phenotypes of OsVQ13-Overexpressing Rice Plants

**Figure 2:**
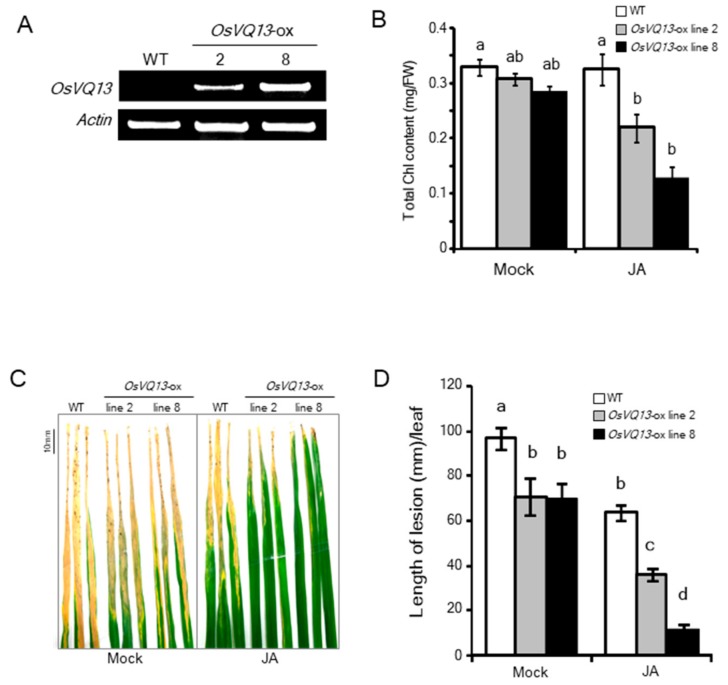
Phenotype of *OsVQ13*-overexpressing rice plants. (**A**) RT–PCR analysis of *OsVQ13* and *actin* expression in wild-type (WT) and *OsVQ13*-overexpressing rice plants (*OsVQ13*-ox; lines 2 and 8). (**B**) Total chlorophyll content in leaf blades after treatment with 100 µM of JA for 3 d in WT and *OsVQ13*-ox rice plants. Values are means ± SE. Data were analyzed using Tukey’s HSD test (*n* = 4 for each genotype). Bars with different letters are significantly different at *p* < 0.05. (**C**) Disease symptoms of rice bacterial blight in WT and *OsVQ13*-ox with mock or 100 μM of JA pretreatment for 24 h. The fifth leaf blades were photographed 14 d after inoculation with *Xoo*. (**D**) The length of lesions on the fifth leaf blades at 14 d after inoculation with *Xoo* with pretreatment with 100 µM of JA for 24 h. Values are means ± SE. Data were analyzed using the Tukey–Kramer test (*n* = 12 for both WT mock and JA; *n* = 7 for line 2 mock; *n* = 12 for line 2 JA; *n* = 10 for line 8 mock; *n* = 12 for line 8 JA). Bars with different letters are significantly different at *p* < 0.05.

We generated *OsVQ13*-overexpressing (*OsVQ13*-ox) rice plants (lines 2 and 8) and confirmed the expression of the transgene through RT-PCR (Figure 2A). To identify JA responses in these transgenic rice plants, we measured chlorophyll (Chl) contents after JA treatment, because it is known that JA promotes a Chl degradation in rice [13]. The Chl contents of *OsVQ13*-ox rice plants were significantly reduced at three days after JA treatment (Figure 2B). 

To determine whether OsVQ13 is involved in JA-mediated resistance to *Xoo*, we performed a resistant test on these transgenic rice plants. The JA-treated or untreated rice plants were inoculated with a virulent *Xoo*, and the length of any blight lesions was measured 14 days after inoculation. The lengths of blight lesions in the *OsVQ13*-ox plants were significantly shorter than those in the wild-type (WT) plants without JA treatment (Figure 2C,D). Furthermore, JA-induced resistance was enhanced in the transgenic rice plants compared to the WT plants (Figure 2C,D).

### 2.3. Identification of OsVQ13-Associating Proteins 

**Figure 3:**
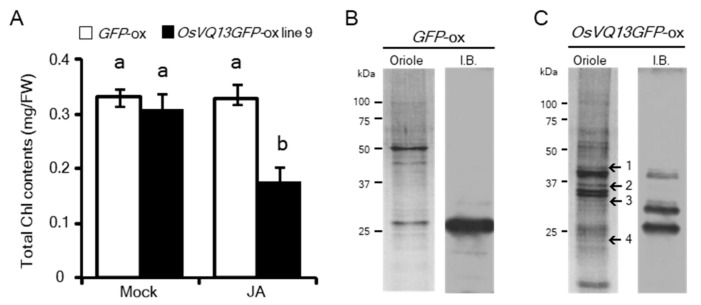
Identification of OsVQ13-associating proteins. (**A**) Confirmation of JA-hypersensitive phenotype in *OsVQ13GFP*-ox plant. Total chlorophyll content in leaf blades after treatment with 100 µM of JA for 3 d in WT and *OsVQ13GFP*-ox rice plants. Values are means ± SE. Data were analyzed using Tukey’s HSD test (*n* = 4 for each genotype). Bars with different letters are significantly different at *p* < 0.05. (**B**,**C**) The proteins co-purified with GFP-Trap from *GFP*-overexpressing (*GFP*-ox: (**B**)) and *OsVQ13GFP*-overexpressing rice plants (*OsVQ13GFP*-ox: (**C**)) were separated through SDS-PAGE, and protein bands were visualized by Oriole straining (Oriole). GFP (**B**) and OsVQ13GFP (**C**) proteins were detected using anti-GFP antibody (I.B.). The numbers on the left indicate the position of the protein size markers in kDa. The putative molecular weight of each protein was as follows: GFP, 27 kDa; OsVQ13GFP, 41 kDa. The numbers on the right of the Oriole staining lane in (**C**) indicate protein bands excised for MALDI-TOF MS protein identification. (**C**) The gel is representative of five independent experiments.

*Arabidopsis* VQ proteins act as positive or negative regulators through interactions with various proteins in response to abiotic or biotic stresses [31]. To determine whether OsVQ13 associates with uncharacterized proteins in rice, we performed a co-immunoprecipitation assay on anti-GFP antibodies derived from *OsVQ13GFP*-ox rice plants. The *OsVQ13GFP*-ox rice plant exhibited a JA-hypersensitive phenotype similar to that of the *OsVQ13*-ox transgenic plant, indicating that the OsVQ13GFP protein was functional in the rice plant (Figure 3A). The *GFP*-ox transgenic rice plant (*GFP*-ox) was used as a negative control. After anti-GFP antibody precipitation, numerous proteins were detected in the *OsVQ13GFP*-ox rice plant sample, whereas only a small number of proteins were detected in the *GFP*-ox rice plant sample (Figure 3B,C). Experiments using *OsVQ13GFP*-overexpressing rice plants were repeated five times, and ultimately, the four proteins that were reproducibly detected at least twice in the immunoprecipitates were selected (Figure 3C). These selected proteins were analyzed using matrix-assisted laser desorption/ionization time-of-flight mass spectrometry (MALDI-TOF MS) and identified in a MASCOT database. As a result, one mitogen-activated protein kinase (MAPK), OsMPK6, and three TFs, namely OsNAC5, OsERF36, and OsMADS2, were identified (Table 1). 

**Figure 4:**
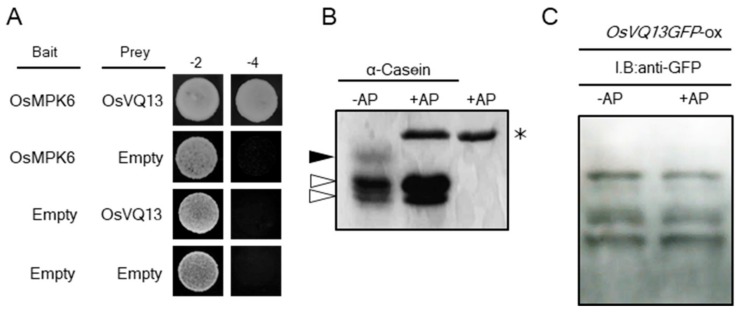
Phosphorylation assay of OsVQ13. (**A**) Interaction between OsVQ13 and OsMPK6 using yeast cells. The yeast AH109 strain was dropped on synthetic dropout (SD) glucose medium lacking Leu and Trp (−2) or on SD glucose medium lacking Ade, His, Leu, and Trp (−4). (**B**) Phosphorylation assay of the α-casein protein through Phos-tag^®^ PAGE. Here, α-casein was a control protein treated with alkaline phosphatase (+AP) or untreated (-AP) (as indicated), separated through Phos-tag^®^ SDS-PAGE. The black arrowhead indicates phosphorylated-α-casein, and the white arrowheads indicate dephosphorylated α-caseins. An asterisk indicates an AP protein. (**C**) Phosphorylation assay of the OsVQ13 protein through Phos-tag^®^ PAGE. Purified OsVQ13GFP proteins were treated with AP (+AP) or were untreated (−AP). The treated proteins were separated through Phos-tag^®^ SDS-PAGE and were detected using anti-GFP antibodies.

Among the OsVQ13-associating proteins, we focused on OsMPK6, which is known to be involved in the rice defense response [32,33]. We first found a direct interaction between OsVQ13 and OsMPK6 proteins using a yeast two-hybrid system (Figure 4A). We next performed Phos-tag^®^ SDS-PAGE to reveal whether OsVQ13 in rice was phosphorylated. In SDS-PAGE using Phos-tag^®^ acrylamide, phosphorylated proteins are trapped by the Phos-tag^®^ sites, which leads to a delay in their migration and a resulting separation from unphosphorylated proteins. This makes it straightforward to identify the phosphorylated proteins from their observed positions on blots [34,35]. As a positive control, we used a commercially available α-casein, which contains both phosphorylated and unphosphorylated forms. When the α-casein was separated by Phos-tag^®^ SDS-PAGE, a slow-migrating band was detected (Figure 4B). The band disappeared following treatment with protein phosphatase, indicating that the upper band was a highly phosphorylated form of α-casein, which could be distinguished from the unphosphorylated form through Phos-tag^®^ SDS-PAGE (Figure 4B). In the case of the sample from the *OsVQ13GFP*-ox rice plant, however, there was no change in the banding pattern detected by anti-GFP antibodies between samples treated and not treated with phosphatase (Figure 4C).

### 2.4. OsVQ13 Affected OsMPK6-Mediated Signaling Pathways in Rice 

**Figure 5:**
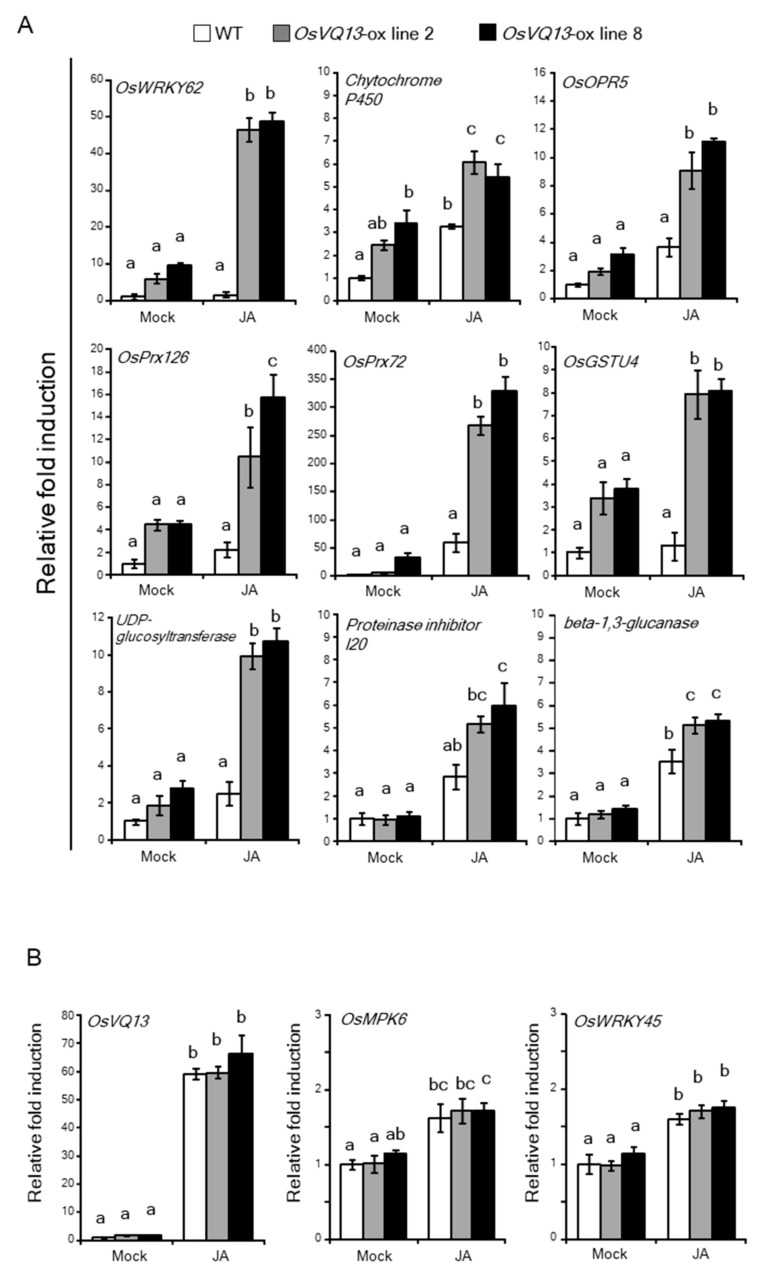
Expression of OsWRKY45-responsive defense-related genes in *OsVQ13*-overexpressing rice plants. (**A**) Expression levels of OsWRKY45-responsive genes after JA treatment. The OsWRKY45-responsive genes identified by Nakayama et al. [4] were used: *OsWRKY62*, *cytochrome P450*, *OsOPR5*, *OsPrx126*, *OsPrx72*, *OsGSTU4*, *UDP-glucosyltransferase*, *Proteinase inhibitor I20*, and *beta-1,3-glucanase*. (**B**) Expression levels of endogenous *OsVQ13*, *OsMPK6*, and *OsWRKY45* after JA treatment. (**A**,**B**) Values are means ± SE. Data were analyzed using Tukey’s HSD test (*n* = 4 for each genotype). Bars with different letters are significantly different at *p* < 0.05.

OsMPK6 is involved in the rice defense response through the phosphorylation of OsWRKY45, a TF that, in turn, plays a crucial role in defense responses in rice [2,4,36,37]. To investigate whether OsVQ13 affects the OsWRKY45-dependent pathway via its association with OsMPK6, we compared the expression levels in WT and *OsVQ13*-ox rice plants of the following OsWRKY45-responsive genes (identified by Nakayama et al. [4]): *OsWRKY62*, *cytochrome P450*, *OsOPR5*, *OsPrx126*, *OsPrx72*, *OsGSTU4*, *UDP-glucosyltransferase*, *Proteinase inhibitor I20*, and *beta-1,3-glucanase*. Genes *OsWRKY62*, *cytochrome P450*, *OsOPR5*, *OsPrx126*, *OsPrx72*, *OsGSTU4*, and *UDP-glucosyltransferase* tended to be upregulated in *OsVQ13*-ox rice plants compared to WT plants in the absence of JA. The expression levels of all genes tested were significantly higher in JA-treated *OsVQ13*-ox rice than in WT plants (Figure 5A). In contrast, the expression levels of endogenous *OsVQ13*, *OsMPK6*, and *OsWRKY45* in *OsVQ13*-ox plants with or without JA treatment were not significantly different from those in WT rice plants (Figure 5B). 

**Figure 6:**
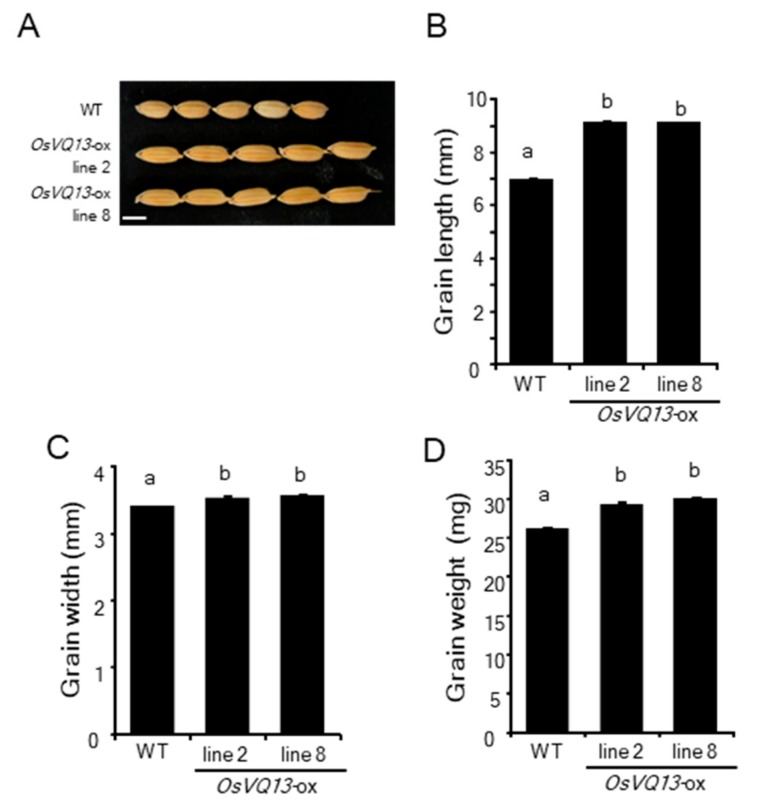
Grain size of *OsVQ13*-overexpressing rice plants. (**A**) Mature grains of the WT and *OsVQ13*-overexpresing rice plants (*OsVQ13*-ox: lines 2 and 8). Bar = 5 mm. The average grain length (**B**), width (**C**), and grain weight (**D**) of the WT and *OsVQ13*-ox plants. Values are means ± SE. Data were analyzed using Tukey’s HSD test (*n* = 100 for each genotype). Bars with different letters are significantly different at *p* < 0.05.

It was also shown that the length, width, and weight of *OsVQ13*-ox grains were significantly larger than WT grains (Figure 6A–D). OsMPK6 has been shown to positively regulate grain size and weight in rice [38,39]. 

## 3. Discussion

We previously demonstrated that the gene expression patterns in response to JA are broadly divided into two phases, the early (<12 h) and late (>12 h) phases, under our experimental conditions [13]. The expression level of *OsVQ13* reached a maximum at 24 h after JA treatment, indicating that OsVQ13 is a late JA-responsive gene in rice. It has been reported that the basal expression of *OsVQ13* in the resistant rice plants harboring a *Xoo*-resistance gene, *Xa3*/*Xa26*, was significantly higher than in corresponding susceptible rice plants [30]. When *OsVQ13* was overexpressed in rice in the current study, the transgenic plants exhibited a JA-hypersensitive phenotype and increased basal and JA-induced resistances against *Xoo*. The increased JA-induced resistance against *Xoo* in transgenic plants depended on the expression levels of the *OsVQ13* transgene. These results indicated that OsVQ13 acted as a positive regulator of JA signaling in rice.

**Figure 7:**
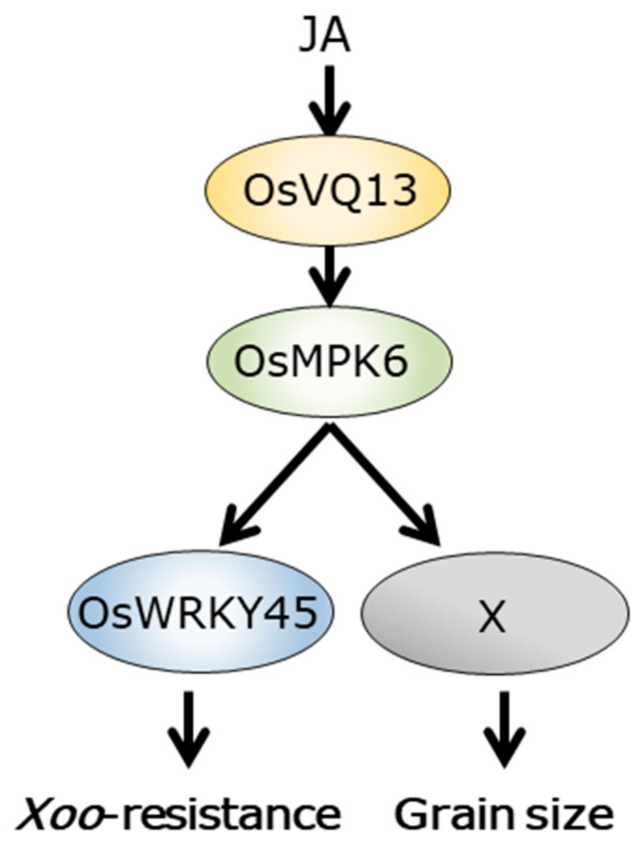
A model for OsVQ13/OsMPK6 complex-mediated signaling pathways in rice. OsVQ13 associates with OsMPK6 and acts as an activator of the OsWRKY45-dependent signaling pathway, contributing to resistance to *Xanthomonas oryzae* pv. *oryzae*. OsVQ13 also affects rice grain development by associating with OsMPK6.

In the present study, we revealed that OsVQ13 associated with OsMPK6 and positively regulated OsMPK6-mediated signaling pathways in rice. OsVQ13 possessed putative phosphorylation sites through MAPK. Furthermore, we suggested the possibility that OsVQ13 may not be a substrate of OsMPK6, although further analysis is needed to determine whether OsVQ13 is phosphorylated or not by OsMPK6 in rice. These results suggest that OsVQ13 acts as a regulatory protein toward OsMPK6, but not as a substrate for OsMPK6. Furthermore, expression patterns of *OsMPK6* and *OsWRKY45* were similar in WT and *OsVQ13*-ox rice plants, suggesting that OsVQ13 positively regulated the expression of OsWRKY45-responsive genes by activating OsMPK6 at the protein level but not at the transcriptional level. The activity of OsMPK6 is suppressed via interaction with the MAPK phosphatase OsMKP1, which dephosphorylates both serine/threonine and tyrosine residues of MAPKs in rice [40]. Thus, it is possible that OsVQ13 blocks the interaction between OsMPK6 and OsMKP1 to activate the OsMPK6–OsWRKY45 cascade in response to JA. Regulation of OsWRKY45 activity via the phosphorylation mediated by OsMPK6 has been analyzed in detail [37]. OsWRKY45 is phosphorylated at Thr266, Ser294, and Ser299 by OsMPK6. Phosphorylation of Ser294 and Ser299 is required for the full activation of OsWRKY45 in the defense response in rice [37]. Conversely, phosphorylation of Thr266 negatively affects the defense response in rice [41]. In the current study, it was demonstrated that OsVQ13 positively regulated the OsWRKY45-dependent signaling pathway by associating with OsMPK6, although there was no direct evidence for the phosphorylation of OsWRKY45 by OsVQ13 in rice (Figure 7). Further studies are needed to test this hypothesis.

Both OsMPK6 and OsWRKY45 act as central regulators of SA signaling in rice [2,4,37]. In the current study, we demonstrated that the expression of some OsWRKY45-responsive genes was upregulated by JA, which also increased their upregulation in *OsVQ13*-ox rice plants. The expression of *OsMPK6* and *OsWRKY45* is upregulated through JA treatment [42,43]. Another important fact is that the expression of *OsVQ13* is also upregulated by BTH, an analog of SA [2]. These results suggest that OsVQ13 plays a critical role as an activator of the OsMPK6–OsWRKY45-dependent cascade involved in both JA- and SA-induced resistance against *Xoo*. 

Generally, SA confers resistance against biotrophic and hemibiotrophic pathogens, whereas JA confers resistance against necrotrophic pathogens in plants. The relationship between JA and SA is antagonistic in many plants. As an example of an *Arabidopsis* VQ protein, plants exhibiting RNA interference (RNAi) of *JAV1* exhibited increased resistance against the necrotrophic pathogen *Botrytis cinerea* [25]. The *Arabidopsis mks1* mutant exhibited increased susceptibility to the biotrophic pathogen *Pseudomonas syringae* [27]. However, in rice, it is unclear whether this antagonistic cross-talk between JA- and SA-dependent defense signaling occurs. In addition to the current study, other studies have demonstrated that the JA-dependent signaling pathway has an important role in resistance against hemibiotrophic pathogens in rice. Accumulations of JA-isoleucine (JA-Ile), a bioactive form of JA that is induced through inoculation with *P. oryzae* [44], and the jasmonate-deficient rice mutants *cpm2* and *hebiba*, exhibited decreased resistance to an avirulent *P. oryzae* [45]. The *osjar1-2* mutant, which exhibits diminished JA-Ile accumulation in leaves, also showed decreased resistance to *P. oryzae* [46]. Expression of the microRNA *miR319b* was upregulated through inoculation with virulent *P. oryzae*. The upregulation of *miR319b* caused the suppression of its target gene, TEOSINTE BRANCHED1/CYCLOIDEA/PROLIFERATING CELL FACTOR1 (TCP)21 (*OsTCP21*). OsTCP21 acts as a positive TF regulating the JA-biosynthetic genes *lipoxygenase2* (*OsLOX2*) and *OsLOX5* [47]. On the other hand, *P. oryzae* converts JA into 12OH-JA by secreting a monooxygenase, Abm. The 12OH-JA suppresses the induction of JA-mediated defense responses [48]. It has been reported that 313 BTH-upregulated genes were identified by microarray analysis in rice [2]. When the expression levels of BTH-upregulated genes were compared to those of JA-responsive genes, as identified by microarray analysis in rice [10], more than half of the BTH-upregulated genes, including *OsVQ13*, were also upregulated by JA [42]. OsNPR1 is degraded by OsCUL3a to suppress OsNPR1-dependent cell death [8]. The expression of *OsNPR1* is also upregulated by JA [49]. Furthermore, an *oscul3a* mutant exhibited increased resistance to *P. oryzae* and *Xoo* by activating both the JA- and SA-signaling pathways [8]. Taken together, these results strongly indicate that JA and SA signaling can interact coordinately in an induced defense response and that OsVQ13 may be a key factor in the rice defense pathway induced by both JA and SA. 

The overexpression of *OsVQ13* has resulted in larger grain sizes compared to WT. The overexpression of *OsMPK6* or a constitutively active version of *OsMPK6* (*CA-OsMPK6*) has also resulted in significantly larger grains [39]. Furthermore, an *oscoi1b* mutant has exhibited a significantly lower grain weight [50], suggesting that JA has a positive effect on the seed development process. However, there is not enough experimental evidence to discuss the function of OsVQ13 in JA-dependent seed development in rice (Figure 7). Further studies are needed.

Among the OsVQ13-associating proteins, the NAC-type TF OsNAC5 positively regulates some defense-related genes in rice, and the expression of *OsNAC5* responds to both JA and abscisic acid [51]. OsNAC5 is also required to express salt stress tolerance in rice [51]. *OsNAC5*-overexpressing rice plants have exhibited increased salt tolerance, whereas *OsNAC5*-RNAi rice plants have exhibited greater sensitivity than the WT to salt stress [51,52]. Recently, it has been reported that JA plays an important role in salt tolerance in many plant species, including rice [53]. RICE SALT SENSITIVE3 (RSS3) acts as a negative regulator of JA signaling by interacting with OsJAZ9 and OsJAZ11, and the *rss3* mutant exhibits the salt-hypersensitive phenotype [54]. OsNAC5 is also known to be negatively involved in leaf senescence in rice [55], whereas JA has a positive effect on leaf senescence in rice [50,56]. There is no information as to whether OsVQ13 is a senescence-associated gene in rice. Further analyses of OsVQ13-associating proteins such as OsNAC5 may provide new insights into the molecular mechanism of JA-dependent biotic and abiotic stress responses in rice.

## 4. Materials and Methods 

### 4.1. Plant Materials, Jasmonate Treatment, and Bacterial Inoculation

Plant and bacteria growth conditions were as previously described, they are grown and cultured in our laboratory by ourselves [15]. The fully opened fifth leaf blades of rice plants were inoculated using the clipping inoculation technique [57]. The lengths of blight lesions on each leaf blade were measured for each leaf at 14 days post-inoculation.

To examine the effects of JA on rice growth and gene expression, rice plants were grown to the four-leaf stage in a growth chamber in Kimura-B liquid medium [58] at 25 °C (24 h light) and then incubated in the same medium supplemented with 100 μM of JA (Sigma-Aldrich, St. Louis, MO, USA). The fourth leaf blades were used for RT-qPCR analyses. 

### 4.2. RT-qPCR

Total RNA was extracted from rice leaf blades from plants of different genotypes and treatments using Trizol (Thermo Fisher Scientific, Waltham, MA, USA) according to the manufacturer’s instructions. RT-qPCR was performed as previously described [15,59]. The sequences of the gene-specific primers used in this study are presented in the Supplementary Table S1.

### 4.3. Construction of OsVQ13-Overexpressing Vectors and Rice Transformation

The *OsVQ13* cDNA (accession number: AK109228) was ligated into the pBI333-EN4 vector [60]. The pBI333-EN4-GFP binary vector was prepared by subcloning the GFP coding sequence into the pBI333-EN4 binary vector. The production of transgenic rice plants was performed as previously described [61,62]. Second- or third-generation plants were used for the experiments. RT-PCR was performed using the OneStep RT-PCR kit (QIAGEN, Hilden, Germany) according to the manufacturer’s instructions. The sequences used for RT-PCR were as follows: *OsVQ13F*, 5′-GACATGTTCGACTACGCGTC-3′ and t*NOSR*, 5′-GTATAATTGCGGGACTCTAATC-3′; *GFPR,* 5′-GCATGGCGGACTTGAAGA-3′; *actin*, forward, 5′-CCTGGAATCCATGAGACCAC-3′ and reverse, 5′-ACACCAACAATCCCAAACAGAG-3′. 

### 4.4. Chlorophyll Content Measurement

Leaf blades treated with 100 μM of JA for four days were homogenized in 1 mL of 80% acetone and centrifuged at 15,000 rpm for 10 min. The specific chlorophyll content was determined as described by Arnon [63].

### 4.5. Subcellular Localization Assay

Seven-day-old rice seedlings were incubated at 25°C in CHU (N6) medium (Duchefa Biochemie, Haarlem, The Netherlands) with 50 mg/L of hygromycin. The GFP signal in the root tissue overexpressing *OsVQ13-GFP* was visualized by using a KEYENCE BIOREVO BZ-9000 fluorescence microscope (Keyence, Osaka, Japan). We used BZ filter GFP (excitation wavelength: 470/40 nm; absorption wavelength: 535/50 nm; dichroic mirror wavelength: 495 nm) and BZ filter DAPI (excitation wavelength: 360/40 nm; absorption wavelength: 460/50 nm; dichroic mirror wavelength: 400 nm). The root tissues were stained with 4′,6-diamidino-2-phenylindole (DAPI) solution (Dojindo, Kumamoto, Japan).

### 4.6. Identification of OsVQ13-Associating Proteins

#### 4.6.1. In Vivo Purification of the OsVQ13GFP Protein

Rice leaf blades (2 g, fresh weight) were homogenized with liquid nitrogen, and the powder was resuspended in 30 mL of extraction buffer 1 (0.4 M sucrose, 10 mM Tris-HCl (pH 7.5), 5 mM β-mercaptoethanol (β-ME), 0.1 mM phenylmethylsulfonyl fluoride (PMSF), and 1% plant protease inhibitor cocktail (Sigma-Aldrich)). After filtration of the slurry through Miracloth (Merck, Darmstadt, Germany), the filtrate was centrifuged (2880× *g*, 20 min, 4 °C). The pellets containing nuclei were resuspended in 1 mL of extraction buffer 2 (0.25 M sucrose, 10 mM Tris-HCl (pH 7.5), 10 mM MgCl_2_, 1% Triton X-100, 5 mM β-ME, 0.1 mM PMSF. and 1% plant protease inhibitor cocktail) and collected by centrifugation (12,000× *g*, 10 min, 4 °C). The pellets were resuspended in 300 µL of extraction buffer 3 (1.7 M sucrose, 10 mM Tris-HCl (pH 7.5), 0.15% Triton X-100, 2 mM MgCl_2_, 5 mM β-ME, 0.1 mM PMSF, and 1% plant protease inhibitor cocktail), and the resuspended pellets were overlaid onto 300 µL of extraction buffer 3. The nuclear proteins were collected by centrifugation (16,000× *g*, 60 min, 4 °C) and suspended in nuclear lysis buffer (10 mM Tris-HCl (pH 7.5), 0.2 mM EDTA, 150 mM NaCl, 0.1% Triton X-100, 5 mM β-ME, and 1% plant protease inhibitor cocktail).

#### 4.6.2. Co-Immunoprecipitation and Protein Gel Blot Analysis

The nuclear proteins were mixed with magnetic bead-conjugated GFP-Trap (Chromotek, Munich, Germany) and incubated overnight at 4 °C. The beads were then washed five times in 0.5 mL of wash buffer II (Chromotek, Munich, Germany). The binding proteins were eluted with sample buffer. The extracted proteins were separated by SDS-PAGE and transferred to a nitrocellulose membrane through semidry blotting. A protein gel blot analysis was performed as previously described [13]. We used anti-GFP (1:1000) and antirabbit IgG horseradish peroxidase-conjugated secondary antibodies (1:20,000).

#### 4.6.3. Protein Identification Using TOF MS

The proteins that were purified by GFP-Trap were separated by 12% SDS-PAGE and stained with Oriole Fluorescent Gel Stain (Bio-Rad, Hercules, CA, USA) for 90 min. The stained protein bands were excised from the gels, washed twice with 100 mM of NH_4_HCO_3_ containing 30% acetonitrile (Wako, Osaka, Japan), washed with 100% acetonitrile, and then dried in a vacuum concentrator. The gels were reductively alkylated with 10 mM of dithiothreitol (DTT) in 100 mM of NH_4_HCO_3_ for 60 min at 56 °C and with iodoacetamide in 100 mM of NH_4_HCO_3_ for 30 min at 25 °C. The gels were washed with 100 μL of 100-mM NH_4_HCO_3_ for 10 min and dehydrated through the addition of acetonitrile. The dried gels were treated with 2 μL of 0.5 μg/μL of trypsin (Promega, Madison, WI, USA) in 50 mM of NH_4_HCO_3_ and incubated at 37 °C for 12 h. Peptides were extracted with 50 mM of NH_4_HCO_3_ in 1% trifluoroacetic acid and 50% acetonitrile. Sample preparation for MALDI analysis was according to the method of Shevchenko et al. [64]. Peptides of proteins that associate with OsVQ13 were identified through MALDI-TOF MS analysis with a Voyager-DE STR (Thermo Fisher Scientific, Waltham, MA, USA). Experiments using *OsVQ13GFP*-overexpressing rice plants were repeated five times, and four out of the five stained gels are presented in the Supplementary Figure S1.

#### 4.6.4. Phos-tag^®^ SDS-PAGE

To determine whether OsVQ13 was phosphorylated, we used Phos-tag^®^ SDS-PAGE (NARD Institute Ltd., Amagasaki, Japan). To achieve dephosphorylation, the beads were prepared in 50 µL of 1× alkaline phosphatase buffer (Thermo Fisher Scientific) and incubated with 2 units of fast alkaline phosphatase (Thermo Fisher Scientific) for 2 h. After incubation, the beads were then washed five times in 0.5 mL of extraction buffer, and the binding proteins were eluted with 2× SDS sample buffer.

### 4.7. Yeast Two-Hybrid System

An analysis of protein–protein interactions using a yeast two-hybrid system was performed as previously described [15]. Images of yeast cells were obtained at day 3.

### 4.8. Additional Information

The accession numbers for the genes discussed in this article are as follows: OsVQ13 (AK109228), OsMPK6 (AK111942), OsWRKY45 (AK066255), OsWRKY62 (AK067834), cytochrome P450 (AK072220), OsOPR5 (AK104193), OsPrx126 (AK061206), OsPrx72 (AK067416), OsGSTU4 (AK103453), UDP-glucosyltransferase (AK064395), proteinase inhibitor I20 (AK105387), and beta-1,3-glucanase (AK068247).

## Figures and Tables

**Table 1:** The list of proteins co-purified with OsVQ13.

No.	Locus No.	Protein Name	Predicted MW (kDa)	Occurrence/Total No. of Experiments
1	Os06g06090	Mitogen-activated protein kinase 6 (OsMPK6)	45	4/5
2	Os11g08210	NAC domain-containing protein 5 (OsNAC5)	35	3/5
3	Os10g41330	Ethylene response factor 36 (OsERF36)	29	2/5
4	Os01g66030	MADS-box transcription factor 2 (OsMADS2)	24	2/5

OsVQ13 co-IP was performed using *OsVQ13GFP*-overexpressing transgenic rice plants. Experiments were independently performed five times. Nos. 1–4 are as in Figure 3. MW: Molecular weight.

## Supplementary Materials

Supplementary materials can be found at https://www.mdpi.com/1422-0067/20/12/2917/s1.
