# Supplementary material for: Jasmonic Acid-Induced VQ-Motif-Containing Protein OsVQ13 Influences the OsWRKY45 Signaling Pathway and Grain Size by Associating with OsMPK6 in Rice
Source: Int J Mol Sci. 2019 Jun 14;20(12):2917. doi: 10.3390/ijms20122917 (PMC6627515; doi:10.3390/ijms20122917)
Supplement: Supplementary file 1 [file ijms-20-02917-s001.zip › Supplementary Figure S1.pdf]

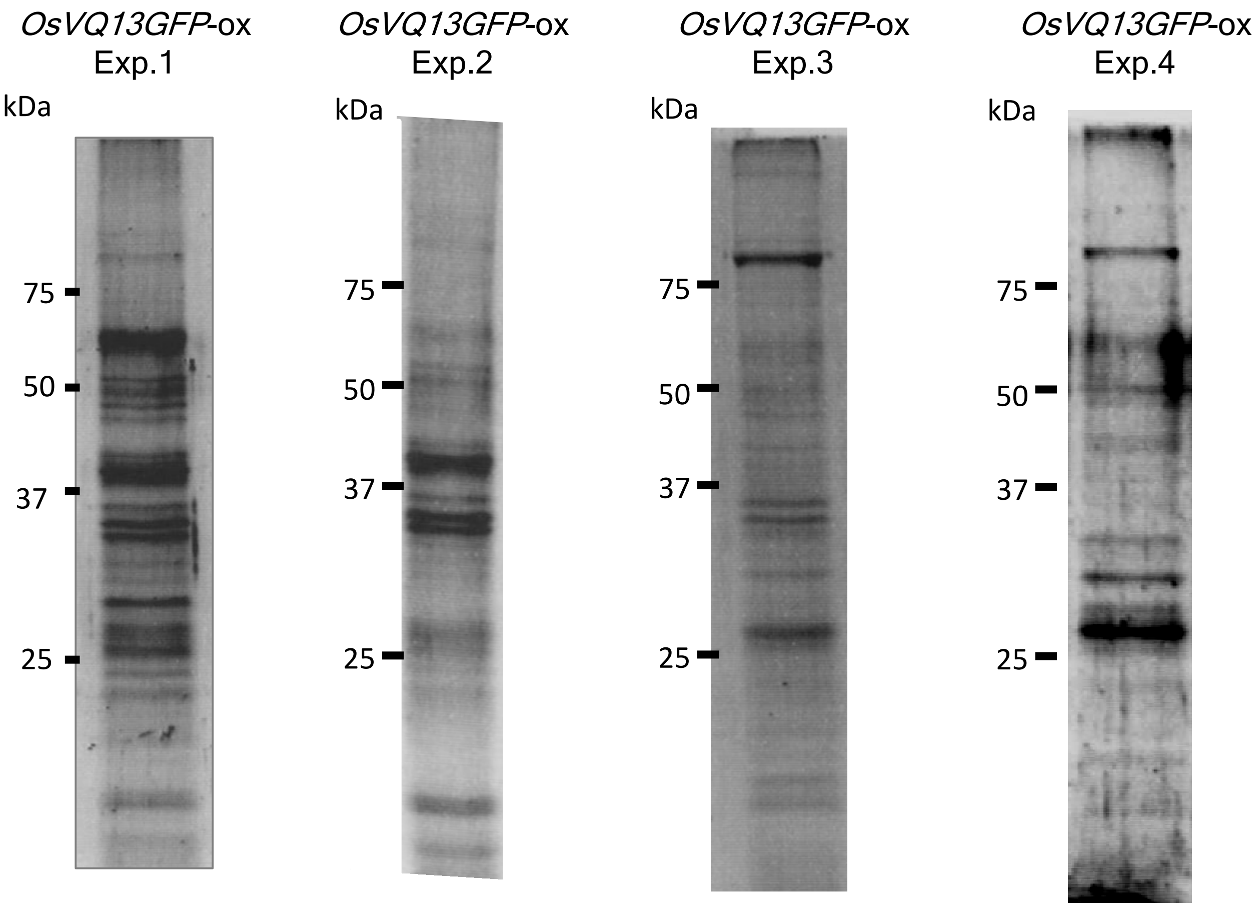

Supplementary Figure S1. Separation of the proteins co-purified with GFP-Trap from *OsVQ13GFP*-overexpressing rice plants (*OsVQ13GFP*-ox) by SDS-PAGE. Protein bands were visualized by Oriole straining. The numbers on the left indicate the position of the protein size markers in kDa. We performed TOF-MS analysis without taking a photograph of the stained gel at the fifth time.
